# Supplementary material for: Aqueous extracts of Urtica dioica (stinging nettle) leaf contain a P2-purinoceptor antagonist—Implications for male fertility
Source: PLoS One. 2022 Jul 28;17(7):e0271735. doi: 10.1371/journal.pone.0271735 (PMC9333203; doi:10.1371/journal.pone.0271735)
Supplement: Attachment — Submitted filename: Response to Reviewers.docx [file pone.0271735.s001.docx]

We have responded to the comments of the Reviewers as follows:

**Reviewer #1:**

Reviewer 1 believed the manuscript to be novel as well as corroborating previous studies identifying P2X1 as a potential target for male contraception. They believed that the data presented would be of interest to readers in the field of P2 purinergic receptors and male fertility and a welcome addition to the literature. We have responded to their comments as follows:

1. Reviewer 1 points out that although stated in the abstract that SN extracts were tested for their effect on prostatic and vas deferens smooth muscle contractility, no data was presented on vas deferens contractility. We have now removed “and vas deferens” from this sentence to avoid this ambiguity (page 2).
2. The Reviewer indicates that we have not definitively shown that the receptor being modulated is of the P2X1 subtype and may be any other P2 purinergic receptor. We agree with the Reviewer but seeing as it is well known in the literature that contractions of the prostate and vas deferens elicited by ATP are mediated by the P2X1 subtype, we have assumed P2X1 to be the receptor being affected in this case. Nevertheless, in accordance with the Reviewer’s suggestion, we have modified the manuscript as follows:
   - The title has been changed to qualify the nature of antagonism from “P2X1” to just “P2” (page 1).
   - Two sentences explaining this concept have been incorporated into the opening paragraph of the Discussion (page 18).
3. As the Reviewer points out, SNLE treatment resulted in reduced testis and bladder weights, while P2X1 KO mice had increased weights of these tissues. This anomaly is difficult to explain but we have now addressed this discrepancy in our discussion (page 19).
4. The labelling of figure 3 and the corresponding caption have been amended in accordance with Reviewer 1’s comments.

**Reviewer #2:**

Reviewer 2 believed the manuscript required some major and minor revisions. We have responded to their comments as follows:

Major revisions:

- Reviewer 2 wishes us to include control data prior to the administration of vehicle or leaf extract to allow the reader to assess the presence or absence of a vehicle effect. Rather than show all of this data, we have stated for all ex vivo tissue experiments that contractions elicited by electrical field stimulation or exogenous administration of agonists “were unaffected by vehicle” implying that the magnitude of contractions was not different before or after incubation with vehicle. This implies that there was no vehicle artefact. A “p value” determined using repeated measures ANOVA is included with each statement so that the reader can assess the degree of statistical significance of any vehicle effect. A statement along these lines was already present in the Results section of the manuscript for the electrical field stimulation experiments (page 12) and we have now added another for the exogenously added agonist experiments (also page 12). We have not revised the manuscript further as we believe that the inclusion of these statements addresses this comment and the addition of five extra graphs would be required to show all of the data. We feel that inclusion of such a large amount of extra data may distract readers from the main message of our paper. However, we are happy to add these graphs as supplementary material if Reviewer 2 feels strongly about including them.
- Reviewer 2 expresses concern about the fast desensitizing nature of P2X1 receptors and suggests that a longer inter-stimulus interval should be tried. This is a valid concern, but we have vast experience with the use of ATP and its analogues to elicit contractile responses of isolated tissues. The choice of inter-stimulus interval chosen in these types of experiments are the result of a balance between the need to avoid desensitisation and the length of time that isolated tissues remain viable in an ex-vivo environment. Over a long period of time (>20 years), we have observed that isolated preparations of rat prostate yield reliably consistent concentration-related contractile responses for approximately 4-6 hours before their viability deteriorates. Consequently, due to the tissue equilibration and drug incubation times needed in our experimental design, we have settled on the 10 min inter-stimulus interval so that the experimental protocol can be completed safely within the tissue viability life span. We also perform laboratory protocols that avoid desensitisation such as washing tissues quickly after a peak contraction is reached and extra tissue washes. Furthermore, we have controlled for any change in tissue sensitivity due to receptor desensitisation by using a before and after treatment protocol alongside a parallel within animal tissue control. As stated in the Results section, parallel tissues produced contractions of similar magnitude and concentration-response curves within tissues did not change over the time course of the experiment following vehicle treatment of tissues. We believe that these observations confirm that receptor desensitisation does not diminish the sensitivity of tissues to ATP or its analogues over the time course of our experiments. A sentence to describe the rationale for these controls has been added to the Methods section (page 8). Using a longer inter-stimulus interval of > 30 min would not allow us to test sufficient concentrations of agonist in our experiment to cover an adequate concentration-response range for proper data analysis. Reviewer 2 also requests clarification on why α,β methylene-ATP should prevent desensitisation. α,β methylene-ATP is a more stable analogue of ATP which causes desensitisation of P2X1-purinoceptors more readily and our use of this agonist was to demonstrate that desensitisation did not occur using our experimental protocol as could be seen with vehicle treatment. No change was made to the manuscript.
- Reviewer 2 suggests that the stinging nettle leaf extract may potentially be acting to inhibit a downstream process to P2X activation instead of as a receptor antagonist. This is again a valid point. However, it is unlikely that the extract is inhibiting a downstream process because P2X1 receptors are ATP ligand gated ion channels that when activated by ATP or one of its analogues open an ionic pore that allows extracellular Ca^2+^ ions to flow into the smooth muscle cell cytoplasm. This influx of Ca^2+^ ions interacts with the smooth muscle machinery directly to produce contraction. An increase in cytoplasmic Ca^2+^ ions is necessary to produce muscle contraction, therefore any downstream inhibitory effects would also inhibit contractile responses to stimulation of other mechanisms such as noradrenergic or cholinergic receptors. This was not observed in our study implying that inhibition of P2X-purinoceptors was the mechanism of action. Furthermore, Reviewer 2 also suggests that direct physiological evidence such as Ca^2+^ imaging or electrophysiology is required to support the claim that the purinoceptor being inhibited is of the P2X1-subtype. However, we believe this not be necessary in this case as it has been largely accepted for many years through immunohistochemical and pharmacological techniques that the only purinoceptor that mediates contractions of the rat prostate smooth muscle is of the P2X1-subtype (Ventura et al, 2003; Br J Pharmacol: 138: 1277-1284). Several sentences have now been incorporated into the Discussion on these points (page 20).
- As Reviewer 1 also mentioned, Reviewer 2 points out that although stated in the abstract that SN extracts were tested for their effect on prostatic and vas deferens smooth muscle contractility, no data was presented on vas deferens contractility. We have now removed “and vas deferens” from this sentence to avoid this ambiguity. Nevertheless, from countless previously published isolated tissue studies, the prostate, vas deferens (and seminal vesicle) of rodents are known to share the same P2X1-purinoceptor contractile mechanism. In order to link our ex vivo findings to our fertility study, a sentence has been added to the Results section on page 15.
- Reviewer 2 asked how many males contributed to the resulting pregnancies in figure 3C. This data has now been incorporated into the text in the Results section (page 18).

Minor revisions:

- We agree with Reviewer 2 that various P2X isoforms are expressed in the testis and other male reproductive systems but histological testis examination and sperm counts following stinging nettle leaf extract is a large undertaking that is beyond the scope of this study. Nevertheless, we have added a comment in the Discussion section related to the possibility that leaf extract may be acting at these sites (page 19).
- The Reviewer is correct that this heading is misplaced and it has been moved to the correct position (page 14, line 352) as pointed out by the Reviewer. We thank Reviewer 2 for pointing out this error on our part.
- The discrepancy in the author list has been amended.

We thank the two Reviewers for their insightful and intuitive comments as we feel that the changes we have made in response to their comments have enhanced our manuscript.
